# Marine microalgae commercial production improves sustainability of global fisheries and aquaculture

**DOI:** 10.1038/s41598-018-33504-w

**Published:** 2018-10-10

**Authors:** Colin M. Beal, Léda N. Gerber, Supis Thongrod, Wutiporn Phromkunthong, Viswanath Kiron, Joe Granados, Ian Archibald, Charles H. Greene, Mark E. Huntley

**Affiliations:** 1 B&D Engineering and Consulting LLC, 7419 State Hwy 789, Lander, WY 82520 United States; 2 University of Hawaii at Hilo, Pacific Aquaculture & Coastal Resources Center, College of Agriculture, Forestry, and Natural Resource Management, 200 W. Kawili St., Hilo, HI 95720 United States; 3 Thai Union Feedmill Co., Ltd., 89/1 Moo2, Tambon Kalong, Amphoe Muang, Samutskorn Province 74000 Thailand; 4 Prince of Songkla University, Department of Aquatic Science, Faculty of Natural Resources, Hat Yai, Songkhla Province 90112 Thailand; 5 Nord University, Faculty of Biosciences and Aquaculture, 8049 Bodo, Norway; 6 Cinglas Ltd., Chester, United Kingdom; 7 Cornell University, Department of Earth and Atmospheric Sciences, 4120 Snee Hall, Ithaca, NY 14853 United States; 8 Cornell University, Department of Animal Sciences, 149 Morrison Hall, Ithaca, NY 14853 United States

## Abstract

A method is described for saving 30% of the world fish catch by producing fishmeal and fish oil replacement products from marine microalgae, the natural source of proteins and oils in the marine food web. To examine the commercial aspects of such a method, we adapt a model based on results of microalgae production in Hawaii and apply it to Thailand, the world’s fourth largest producer of fishmeal. A model facility of 111 ha would produce 2,750 tonnes yr^−1^ of protein and 2,330 tonnes yr^−1^ of algal oil, at a capital cost of $29.3 M. Such a facility would generate $5.5 M in average annual net income over its 30-year lifetime. Deployment of 100 such facilities in Thailand would replace all domestic production of fishmeal, 10% of world production, on ~1.5% of the land now used to cultivate oil palm. Such a global industry would generate ~$6.5 billion in annual net income.

## Introduction

Fishmeal and fish oil are unique nutritional ingredients, produced by rendering ~30% of the wild fish catch. Annual production has been limited since the 1980s, when global fish catch reached maximum sustainable yield, at 5-6 million tonnes fishmeal and 1 million tonnes fish oil^1^.

Demand has been increasing, especially as an essential ingredient in aquafeeds. Fishmeal offers a high-protein (60–65%) ingredient, with a balanced amino acid profile. Fish oil has a high level of n-3 highly unsaturated fatty acids (HUFA), which promote optimal growth and health. Prices of both commodities have more than tripled in the past 10 years. The aquaculture industry is the fastest-growing sector of food production in the world, growing at 8.8% per year from 1980 to 2010^2^.

The question is, where will fishmeal and fish oil come from in the future? The present supply is unsustainable. Replacements have been sought, but no satisfactory replacement products exist. The best alternate sources of protein currently available - soybean protein concentrate, wheat gluten, or corn gluten - still need to be supplemented with essential amino acids like methionine and lysine^3^. Plant protein meals also contain anti-nutritional components which compromise digestion^4^. Replacements for fish oil are more problematic, as direct sources of n-3 HUFA are not produced in sufficient quantities by terrestrial plants^5^.

The best sources of protein and oil for the diets of marine animals are marine microalgae - the very base of the marine food chain. Marine microalgae have a balanced amino acid profile, and some of them are the natural source of n-3 HUFA. Microalgae would be a commercial replacement for the highest quality fishmeal and fish oil^3,6^, but the cost of production has been considered too high.

This study builds on recent advancements in large-scale algae productivity that demonstrate average yields of 78 tonnes ha^−1^ yr^−1^ ^7^. The algae produced by Huntley *et al*.^7^ were separated by solvent extraction into an oil fraction for fuels and a protein-rich (63%) algae meal (as described by Beal *et al*.^8^) used in feeding trials with shrimp and other species^9,10^; the techno-economics of a 111-ha facility, based on the production achieved, was modeled to produce fuels and feeds in the USA^8^.

To examine the commercial viability of a microalgae facility producing replacements for both fishmeal and fish oil, we reconfigure the model for Thailand, the world’s third largest exporter of farmed shrimp^11^. Shrimp feed consumes more fishmeal (~30% of the global total) than any other aquafeed. Thailand now consumes ~5% of global fishmeal just for shrimp feeds. Shrimp feeds also require ~10% of the total global production of fish oil^12^. Production costs are lower in Thailand than in the USA, and thus greater potential exists for profitability. Finally, it makes sense to locate the source of production near the consumer as this reduces transportation and improves product freshness.

## Methods

### System Design

**Figure 1:**
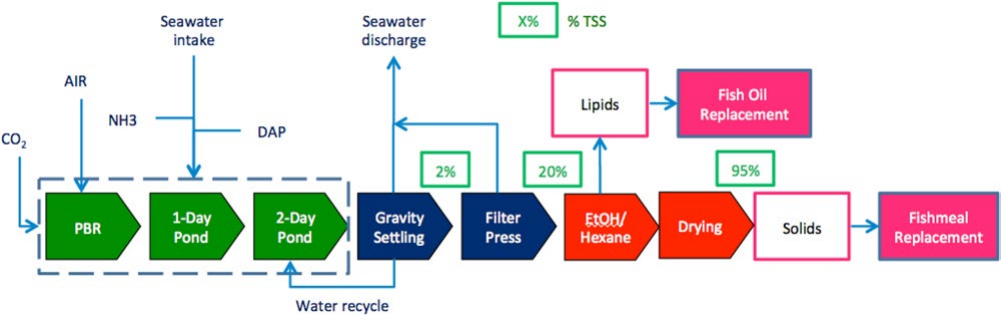
Technology process lineup for fishmeal and fish oil replacement products from algae.

The algae production system modeled in this study (Fig. 1) is based on the cultivation facility presented by Beal *et al*.^8^ and Huntley *et al*.^7^, but is located in Thailand and modified to include a total lipid extraction using ethanol and hexane^13^. The cultivation facility consists of a hybrid system of photobioreactors (PBR) and raceway ponds for cultivation of *Desmodesmus* sp. The facility includes 92 ha of sunlit cultivation area and 114,000 m^3^ of growth volume. Seawater is supplied from a 50-m pipeline and a gravity-based canal system typical of Thailand aquaculture (requiring 0.9 kJ/L for pumping). Unlike the design by Beal *et al*.^8^, volume transfers are accomplished by pumping rather than gravity flow (including pumping of sludge (99 MJ/d), discharge water (2,300 MJ/d), inoculum (1,700 MJ/d), and new seawater (3,100 MJ/d)). Nutrients are provided as ammonia and diammonium phosphate (DAP). Pure carbon dioxide (100%) is purchased as food-grade compressed gas with an uptake efficiency, defined as the amount of carbon assimilated into biomass divided by the amount of carbon supplied to the culture, of 79% in the growth volumes. The algal biomass elemental composition consists of 48% carbon, 6.3% nitrogen, and 0.6% phosphorus with a productivity of 23.8 g/m^2^-d^7^. The biomass contains 39% protein, 37% lipid, 21% carbohydrate, and 3% ash. Daily harvests are conducted with 48,000 m^3^ of growth volume with a modeled algal concentration of 0.46 g/L. Electricity is consumed for circulating the growth media in the PBRs and ponds, transporting carbon dioxide, mixing nutrient tanks, and seawater supply, with a total electrical input of 203 kWh/ha-d. Pond liners (reinforced polypropylene) have a 30-year life, while PBR plastic (polyethylene) is replaced every three months at a cost of $0.47/m^2^. The system has an effective capacity factor of 95% (347 days of operation per year).

The harvesting process consists of natural settling and a filter press. The two-step settling process reported by Beal *et al*.^8^ recovers 94% of the algal biomass to concentration of 20 g/L^8^. A filter press is used to increase concentration to 200 g/L (80% moisture) with 98% recovery efficiency and 1.1 kJ/L of electrical input.

Lipid extraction is conducted according to the methods described by Molina Grima *et al*.^13^, recovering 90% of the total lipids. For each kg of algal biomass, extraction requires 1.26 L of ethanol (with 1% loss), 0.76 L of hexane (with 1% loss), 0.27 L of acetyl chloride (with 1% loss), 221 L of cooling water (with 0.1% loss), 1.5 kg of steam (with 0.1% loss), 5.6 kJ of electricity, and 1.1 kJ of heat for distillation. A ring dryer is used to further dry the recovered biomass to 95% solids, requiring 9.1 MJ/kg of algae. The facility generates 6.7 tonnes of crude lipids and 13.5 tonnes of dry algal biomass per day. The residual algae meal contains 59% protein.

Energy and material flows for the system are presented in Table 1. The life-cycle energy impact for each input and output are taken as global average values from ecoinvent© version 3.2^14^. Prices are based on current market prices in Thailand^15,16^. Greenhouse gas impacts are also sourced from ecoinvent©, version 3.2.

**Table 1:** Energy and material flows. Energy impacts are global averages^14^ and prices are based on Thailand market prices^16,17^.

Inputs	Value (X)	*Energy Equiv*. (*MJ/X*)	Energy Impact (MJ/d)	*Price* (*$/X*)	Cost/Revenue ($/d)	GHG Impact* (g CO_2_e/X)	GHG (g CO_2_e/d)
Cultivation							
Water Supply Electricity (MJ/d)	27,300	*2*.*65*	72,300	*0*.*02*	606	*175*	4,770,000
Volume Transfer Electricity (MJ/d)	7,210	*2*.*65*	19,100	*0*.*02*	160	*175*	1,260,000
PBR Airlift Circulation Electricity (MJ/d)	12,800	*2*.*65*	34,000	*0*.*02*	286	*175*	2,250,000
Pond Circulation Electricity (MJ/d)	33,600	*2*.*65*	89,000	*0*.*02*	746	*175*	5,880,000
Nutrient Stock Tank Mixer (MJ/d)	564	*2*.*65*	1,490	*0*.*02*	12.5	*175*	98,700
Carbon Dioxide Consumed (kg/d)	49,400	*8*.*90*	440,000	*0*.*08*	3,710	*910*	45,000,000
Ammonia Consumed (kg/d)	1,540	*40*.*5*	62,500	*0*.*46*	701	*2090*	3,220,000
DAP Consumed (kg/d)	537	*28*.*7*	15,400	*0*.*41*	221	*1470*	790,000
PBR Plastic (m^2^/d)	3,060	*2*.*01*	6,160	*0*.*47*	1,440	*272*	833,000
		***Cultivation Energy*** (***MJ/d***)	**740**,**000**	***Cultivation Cost*** (***$/d***)	**7**,**880**	**Cultivation GHG** (**g CO**_**2**_**e/d**)	**64**,**100**,**000**
Harvesting							
Pump Secondary Sludge (MJ/d)	26.7	*2*.*65*	70.6	*0*.*02*	0.59	*175*	4,660
Filter Press Operation (MJ/d)	1,110	*2*.*65*	2,950	*0*.*02*	24.8	*175*	195,000
		***Harvesting Energy*** (***MJ/d***)	**3**,**020**	***Harvesting Cost*** (***$/d***)	**25**.**3**	**Harvesting GHG** (**g CO**_**2**_**e/d**)	**200**,**000**
Extraction							
Extraction Electricity (MJ/d)	1,460	*2*.*65*	3,860	*0*.*02*	32.3	*175*	255,000
Ethanol (kg/d)	201	*45*.*9*	9,220	*0*.*44*	87.8	*1090*	219,000
Acetyl Chloride (kg/d)	60.0	*124*	7,420	*0*.*81*	48.4	*7670*	460,000
Extraction Heat (MJ/d)	184,000	*1*.*30*	239,000	*0*.*005*	827	*80*	14,700,000
Solvent Consumed (kg/d)	101	*22*.*3*	2,240	*4*.*43*	446	*320*	32,200
Cooling Water (m3/d)	4.47	*5*.*00*	22.3	*0*.*61*	2.74	*0*.*00*	0
Steam (kg/d)	30.3	*2*.*80*	84.9	*0*.*003*	0.09	*0*.*00*	0
		***Extraction Energy*** (***MJ/d***)	**262**,**000**	***Extraction Cost*** (***$/d***)	**1**,**440**	**Extraction GHG** (**g CO**_**2**_**e/d**)	**15**,**700**,**000**
		***Energy Input*** (***MJ/d***)	**1**,**010**,**000**	***Total Cost*** (***$/d***)	**9**,**350**	**Total GHG** (**g CO**_**2**_**e/d**)	**80**,**000**,**000**
Outputs							
Lipids (kg/d)	6,710	*53*.*1*	356,000	*1*.*80*	12,100	NA	NA
Non-lipid Biomass (kg/d)	13,500	*25*.*1*	339,000	*1*.*40*	18,900	NA	NA
**Total Output for Facility** (**kg/d**)	**20**,**200**	***Energy Output*** (***MJ/d***)	**695**,**000**	***Revenue*** (***$/d***)	**31**,**000**	**Total Algae Yield** (**kg/d**)	**20**,**200**
		***EROI***	**0**.**69**	***Revenue***/***Cost*** (***−***)	**3**.**31**	**GHG per kg Algae** (**kg CO**_**2**_**e/kg**)	**3**.**96**

*GHG impacts not shown for PVC (2,090 g CO_2_e/kg), transport of materials and waste (170 g CO_2_e/tkm), and waste disposal (130 g CO_2_e/kg).

The total capital cost for the facility is $29.3 M (Supplementary Table S1). All capital costs were adjusted to Thailand prices using a geographic cost modifier of 0.58^15–17^ with respect to costs in the U.S. as determined previously by Beal *et al*.^8^. Labor requirements to grow and process the algae (Supplementary Table S2) are based on Beal *et al*.^8^, but adapted to Thailand labor costs^16,17^.

### Techno-economic Assessment

To evaluate the economic feasibility of the integrated system, we calculate the net present value (NPV) for the facility after 30 years of operation using a cumulative discounted cash flow method^8,18,19^. Supplementary Table S3 lists the critical input parameters for the cash flow analysis.

The NPV is calculated as the cumulative discounted cash flow (*DCF*), represented as

1 $$NPV=\sum _{k=0}^{n}\,DC{F}_{k}$$

The discounted cash flow (*DCF*_*k*_) associated with the facility for year *k* is calculated as

2 $$\begin{array}{ccc}{\rm{for}}\,{\rm{k}}=0 & DC{F}_{0}=-\,{C}_{eq} & [\$/{\rm{yr}}]\end{array}$$

where *C*_*eq*_ is the equity portion of the total capital cost (40%) and

3 $$\begin{array}{cc}{\rm{for}}\,{\rm{k}}\ge 1 & DC{F}_{k}=\frac{{R}_{tot}-{C}_{aop}}{{(1+i)}^{k}}\end{array}$$

where *R*_*tot*_ is the annual revenue, *C*_*aop*_ is the annual operating cost, and *i* is the discount rate. Annual operating costs are expressed as

4 $${C}_{aop}={C}_{E\& M}+{C}_{land}+{C}_{mtn}+{C}_{ins}+{C}_{loan}+{C}_{tax}+{C}_{labor}$$

and include energy and materials (*C*_*E*&*M*_), land (*C*_*land*_)^20,21^, maintenance (*C*_*mtn*_), insurance (*C*_*ins*_), loan payments (*C*_*loan*_), taxes (*C*_*tax*_), and labor (*C*_*labor*_). For each year, taxes are calculated as the product of the tax rate (*t*) and the difference between the net income (*NI*) and the losses carried forward (*LF*) from the previous year (if any), represented as

5 $${C}_{tax}=t\cdot (NI-LF)=t\cdot ({R}_{tot}-{C}_{E\& M}-{C}_{land}-{C}_{mtn}-{C}_{ins}-{C}_{labor}-D-I-LF)$$

where *D* is depreciation and *I* is loan interest.

### Energy Return on Investment

The Energy Return on Investment (EROI) is calculated as the ratio of the energy impact of outputs (algal biomass meal and algal lipids) to the energy impact of inputs (all material and energy inputs)^8,22^. Energy impacts for the input/output flows were obtained from ecoinvent© version 3.2^14^, as listed in Table 1.

### Greenhouse Gas Accounting

Life-cycle greenhouse gas (GHG) emissions for the production facility are calculated by applying the GHG impact from ecoinvent© database version 3.2 (IPCC 2013 methodology)^14^ for each material and energy flow (listed in Table 1). Emissions associated with PBR plastic, pond liner, and pipes are included, but those for processing equipment (expected to be negligible) are excluded due to lack of available data. Transport emissions are estimated based on transporting raw materials and waste 100 km. To enable comparisons with other agricultural crops that are used as aquaculture feed ingredients, such as soybeans, the GHG emissions are reported per kg of total algal biomass produced by the facility (i.e., kg CO_2_e/kg algae). The system boundary includes the facility, as well as upstream impacts from material and energy flows. Waste disposal from the facility is also included. Calculating the GHG emissions per unit of algae produced and not per unit of one of the co-products (i.e. algal oil or algal meal) avoids the necessity of performing any controversial allocation of impacts between the two co-products.

### Sensitivity and Uncertainty Analysis

A sensitivity analysis was conducted to understand the individual effects of the main technical parameters on the economic and sustainability performances of the facility. These parameters included: productivity (8.5–42 g/m^2^-d), lipid content (27–47%), efficiency of carbon dioxide absorption (50–95%), lipid extraction efficiency (70–95%), capacity factor (0.8–0.98), and heat for extraction (±30%).

Secondly, an uncertainty analysis was conducted by applying Monte-Carlo probability distributions to the material and energy flows, capital costs, and labor costs for the system. The objective was to understand the combined effect of major technical and economic assumptions subject to uncertainty on the economic and sustainability performances of the facility. The probability distribution functions from Gerber *et al*.^18^ were established for the facility design of Beal *et al*.^8^, and have been used in the present study for the uncertainty analysis. The uncertainty range for the productivity in this simulation has been narrowed to consider only the uncertainty associated with the experimental data of Huntley *et al*.^7^, which would be more representative of a commercial facility built under specific conditions, and not the full range from the literature considered in the sensitivity analysis, that was used to explore the extreme possibilities. For the change in prices from the US case to the Thailand case, the mean value has been adapted to the new location, and the standard deviations for the old locations have been used. Probability distributions for each model parameter are listed in Supplementary Table S4.

### Data

The datasets generated during and/or analyzed during the current study are available from the corresponding author on reasonable request.

## Results

### Techno-economic Results

The NPV of the facility after 30 years of operation was determined to be $26.9 M. This profit represents a 92% return on investment. During the first 10 years of operation, when loan payments and depreciation are applied, the cumulative discounted cash flow increases from −$11.7 M (equity share of capital cost) to $9.5 M. From year 11–30, annual gross revenue ($10.7 M) exceeds annual costs ($4.1 M) by $6.6 M, resulting in $1.3 M of annual tax payments and a steady increase in cumulative discounted cash flow to $26.9 M. The largest contributors to the capital costs ($29.3 M) include pond liner ($8.4 M), pipes ($3.8 M), pumps ($2.8 M), ring dryer ($1.6 M), filter press ($1.2 M), extraction equipment ($1.2 M), and buildings ($1.3 M). When summing other costs over the entire 30-year facility lifetime, the largest costs include: taxes ($45.9 M), carbon dioxide ($38.6 M), PBR plastic ($15.0 M), labor ($11.1 M), electricity for circulating growth volumes ($10.7 M), heat ($8.6 M), loan interest ($8.2 M), insurance ($7.8 M), maintenance ($7.8 M), ammonia ($7.3 M), and water supply electricity ($6.3 M), for a total of $168 M. Revenues from algae oil (fish oil replacement) and residual algal biomass (fishmeal replacement) over the 30-year facility life are $126 M and $197 M, respectively.

The NPV could be increased by negotiating lower tax rates, obtaining low-cost or waste sources of CO_2_, developing longer lasting PBR plastic, implementing automated harvesting methods to reduce labor costs, eliminating pond liners, and using canals to replace pipes. As evaluated in the sensitivity and uncertainty analyses below, profit is dependent on the algal productivity and sale prices of the algae products. If aquafeed prices continue to rise, the algae replacement ingredients could fetch higher prices and generate greater profit. Conversely, if the algae products are valued at current market prices for soy oil ($0.77/kg) and soy meal ($0.30/kg) NPV is reduced to −$21.0 M after 30 years of operation. Similarly, a 50% increase or decrease in algal biomass productivity (baseline of 23.8 g/m^2^-d) would change the NPV to $53.4 M or −$12.7 M, respectively.

### EROI Results

**Figure 2:**
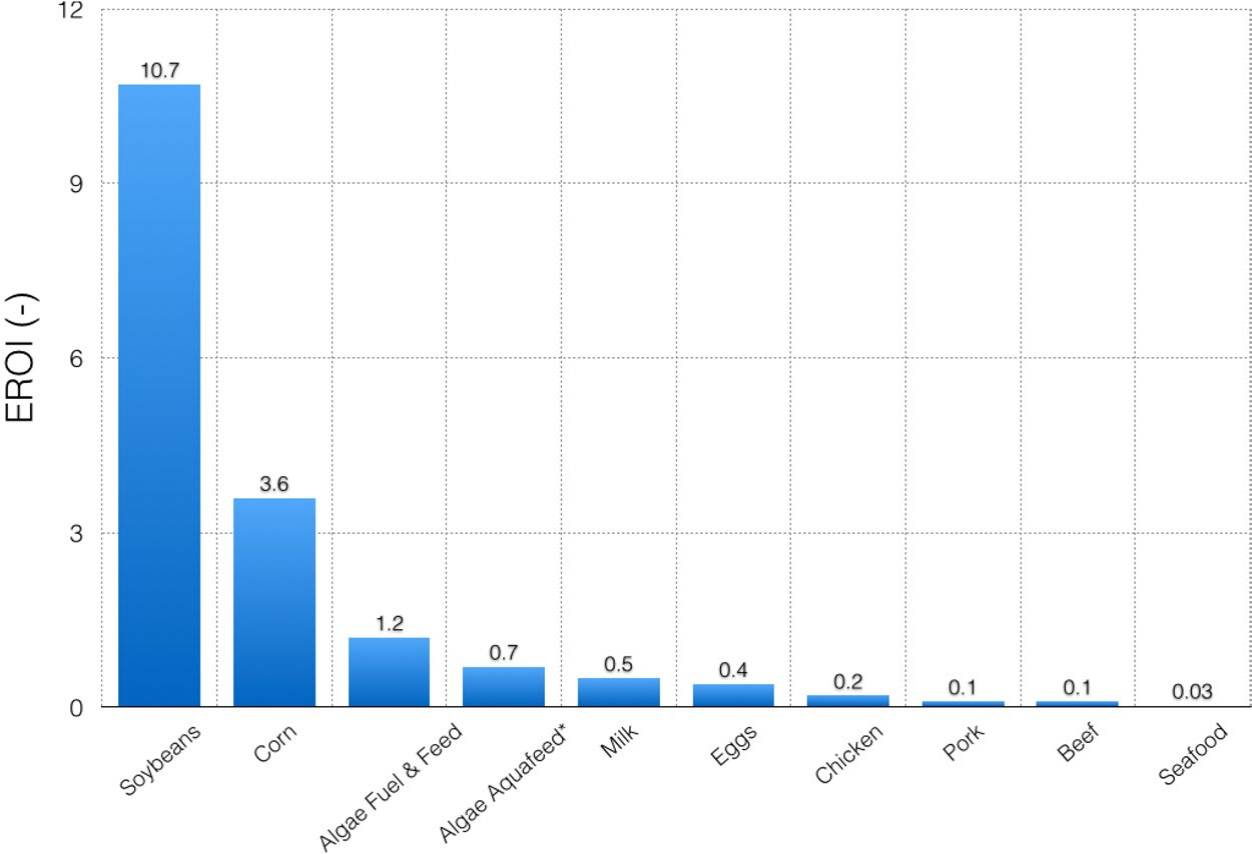
Energy return on investment (EROI) for this study* and a variety of feed and food protein products. Data from [Weidema^14^; Beal^8^; de Vries^32^; USDA^33^; Mitchell and Cleveland^28^].

The EROI for the facility in this study is 0.69 (Tables 1 and 2), which indicates that more energy is expended than generated. The largest energy expenditures are associated with carbon dioxide acquisition (44%), heat for extraction and drying (24%), electricity for pond circulation (9%), and electricity for water supply (7%) (Table 1). Figure 2 presents the EROI for this study in comparison to prior assessments of algae and other protein products. The EROI for this study is of the same order of magnitude as most EROI results for algal biomass production published in the literature^23,24^. For a very similar production system, Beal *et al*.^8,25^ found the EROI to range from 0.34 to 8.35 (with most probable results around 1.2) for 20 independent cases located in either Texas or Hawaii. However, that study assumed co-location with a purified stream of waste carbon dioxide and lower heating requirement for drying, which improves the EROI results as compared to this study. Similarly, Sills *et al*.^24^ show that most prior EROI assessments either assume CO_2_ is sourced from flue gas or exclude the upstream impacts associated with obtaining CO_2_. When the full energetic cost of obtaining carbon is included, the EROI is significantly reduced^25–27^.

**Table 2:** TEA results and life-cycle greenhouse gas emissions for algae production in Thailand for fishmeal and fish oil replacements.

Results	Mean Value	Uncertainty Range
NPV (Year 30)	$26.9 M	−$9.34 M–$40.0 M
EROI	0.69	0.50–0.78
GHG Emissions (kg CO_2_e/kg algae)	3.96	3.29–4.34

The uncertainty ranges are given considering the 5% and 95% quantiles of the Monte-Carlo results.

As shown in Fig. 2, the algal products modeled in this study outperform many other protein sources with respect to EROI. Seafood, in particular, has a very low EROI (0.03)^28^, which indicates that replacing fish oil and fishmeal with algal biomass could provide significant primary energy savings. However, the terrestrial feed crops of corn and soybeans have a significantly higher EROI than the algae products in this study. Unlike algae, terrestrial crops do not require external carbon supply or continuous mixing during cultivation, and the drying requirement is much lower than for algal biomass. As a result, the superior areal productivity of algae in comparison to terrestrial crops is offset by the carbon and energy demands required for production.

### GHG Results

As shown in Tables 1 and 2, the GHG impact of algal biomass in this production model is 3.96 kg of CO_2_e per kg of algal biomass (including oil and meal fractions). Similar to the energy impacts, most GHG emissions are associated with upstream impacts for carbon dioxide (56% of total) and heat produced from natural gas (18% of total). The GHG emissions can be compared with soybeans, which can be considered as a reference scenario. The average GHG impact of soybeans sold on the global market, according to ecoinvent 3.2, is 3.90 kg CO_2_e/kg soybeans. This is comparable to the GHG emissions calculated here for microalgae. However, the global number for soybeans is an average of soybean production in several regions and GHG emissions are highly affected by deforestation rates, resulting in a wide range from 0.39 kg CO_2_e/kg soybean with no deforestation to 5.78 kg CO_2_e/kg soybean with high deforestation. The microalgae would then be environmentally competitive only if substituting soybeans produced in regions with high deforestation rates. Microalgae could be made environmentally more competitive by using electricity and heat from renewable sources, as already discussed in Beal *et al*.^8^, or by developing cultivation systems that could use flue gas from waste streams^29^ instead of pure carbon dioxide that requires large upstream energy inputs.

### Sensitivity and Uncertainty Results

The results for the sensitivity analysis for critical modeling parameters are displayed in Supplementary Figs S1, S2 and S3. For all the three indicators, productivity is the parameter most affected, considering ranges that go from low productivities (8.5 g/m^2^-day) that have been historically achieved in many of the existing large-scale facilities prior to the 23 g/m^2^-day demonstrated in Huntley *et al*.^7^ to high productivities (42 g/m^2^-day) that are theoretically possible but that have not yet been achieved in practice. The EROI and the GHG emissions (see Supplementary Figs S2 and S3) are, however, not heavily impacted even by a very significant change in productivity, and remain in the same order of magnitude, while the NPV (see Supplementary Fig. S1) would become negative at low productivities and more than triple at high productivities. Individual variations in any of the other technical parameters have very little impact on the system performance, except for a lower capacity factor (0.8 instead of 0.95; 292 days of operation rather than 347) that would reduce the NPV by as much as 50%. The reason why the potential variations in lipid content and lipid extraction efficiency have very little influence on the system performance is because of the co-production strategy that valorizes the whole algal biomass as a valuable product either as algal oil or as lipid-extracted algae. This makes the production system more resilient to potential variations that would affect the ratio between produced oil and algal meal.

**Figure 3:**
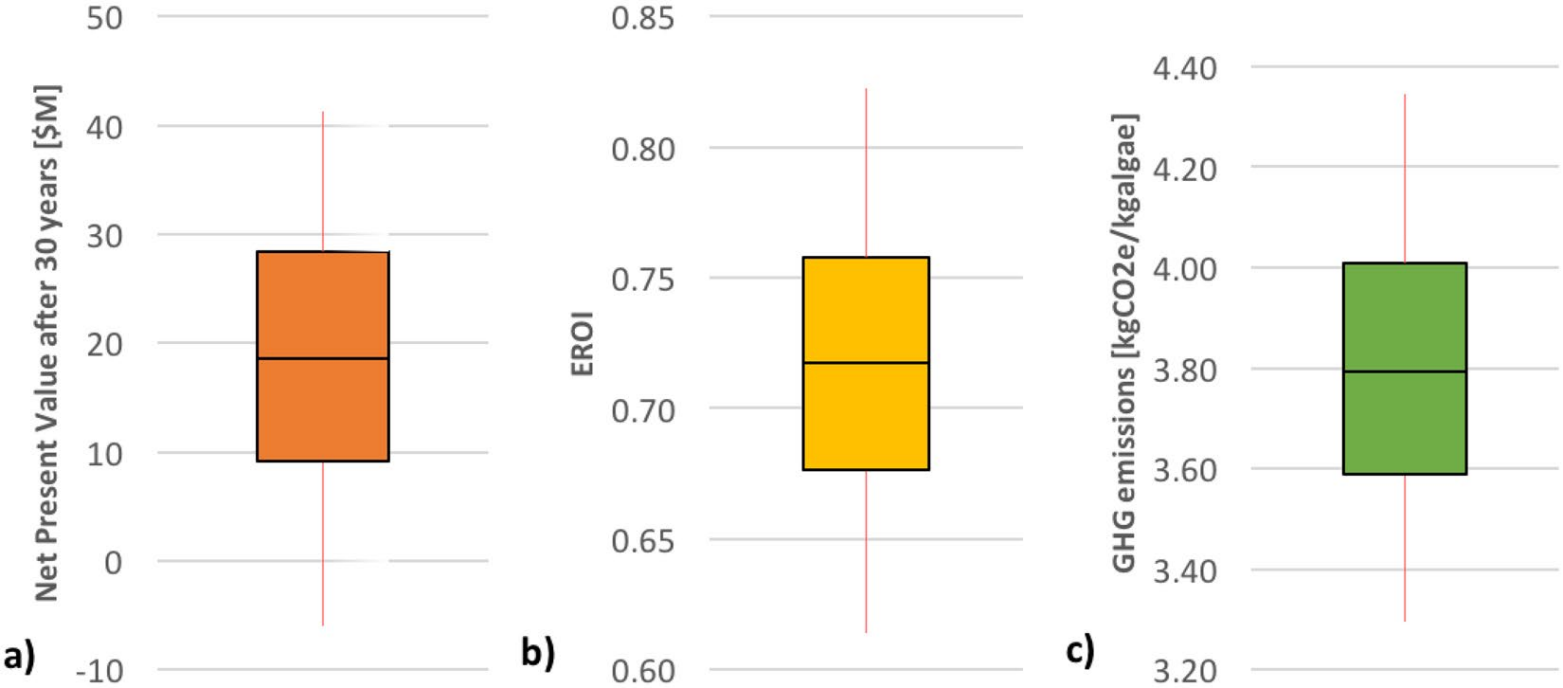
Uncertainty analysis using a boxplot representation for (**a**) the net present value (**b**) the EROI and (**c**) the GHG emissions. The black line in the middle of the colored area represents the median. The inferior and superior limit of the colored area represent the 25% and 75% quantiles, respectively. The inferior and superior limits of the red bars represent the 5% and 95% quantiles, respectively.

Figure 3 shows the results of the uncertainty analysis for combined technical and economic parameters on the NPV (a), the EROI (b), and the GHG emissions (c). The median for the NPV is $15.8 M, meaning that 50% of the simulations were above this value. It does not coincide with the base case NPV of $26.3 M because the mean and the median are not equivalent. More than 75% of the simulations yielded a positive NPV, indicating that the facility has a significant probability of being profitable, except under a combination of multiple unfavorable economic conditions and technical parameters. The EROI (Fig. 3b) and the GHG emissions (Fig. 3c) stay within the same order of magnitude even considering the combined uncertainty. It should be pointed out, however, that the uncertainty analysis did not include the upstream uncertainty associated with the life cycle inventory data used to calculate these indicators, in terms of energy inputs and GHG emissions for the energy and material flows required by the facility, because such data were not available. Only the technical parameters associated with the facility operation and the economic parameters were considered, the latter having no effect on these two indicators.

## Discussion

Thailand annually produces about 0.40–0.50 million tonnes of fishmeal, or about 10% of the world total^30^. At a protein content of 67%, the international standard, that amounts to 0.27–0.35 million tonnes protein. Using microalgae with a 59% protein content, a 111-ha facility such as the one modeled here would produce 2,750 tonnes yr^−1^ protein, or 24.8 tonnes ha^−1^ yr^−1^ protein.

To replace Thailand’s current fishmeal production with algae meal would require from 98 to 127 such facilities, on a total land area about 10,900 to 14,100 ha. One hundred such facilities (11,100 ha) would produce 0.28 million tonnes yr^−1^ of protein, comparable to 0.40 million tonnes yr^−1^ of fishmeal. This amount of land is currently available in Thailand; as of 2016, more than 750,000 ha were under cultivation for oil palm in Thailand. The 11,100 ha required for 100 algae production facilities represent only 1.5% of the land dedicated to oil palm. Such an industry would require a capitalization of $3.0 billion and would yield annual net income of $0.66 billion on annual sales of $1.0 billion.

On a global scale, replacement of fishmeal by algae meal would need about ten times more capital and land, for a total of $30 billion and 111,000 ha. Granted that the algae facilities are not likely to all be located in Thailand, despite the modest amount of land required, we presume that enough land can be found in comparable environments – tropical locations where the cost of capital and labor are comparable to those in Thailand, or perhaps even more favorable. Global net income of $6.5 billion on sales of $10 billion await players in this new industry, which is poised to grow quickly.

Algae production is a far more sustainable industry than continuing to harvest 30% of the world fish catch for fishmeal and fish oil at ever-increasing cost. The release in fishing pressure could have a dramatically favorable effect on marine ecosystems. The fishes that are caught for rendering into fish oil and fishmeal are typically small, primarily herbivorous fish such as anchovies and menhaden. By not fishing for these fish we leave a huge food resource behind that fuels the production of fishes at higher trophic levels, including finfishes like tuna and salmon that are currently limited in supply. This would reverse the trend of fishing down the food web and would go a long way towards restoring sustainable global fisheries^31^.

A viable commercial technology is presented for producing marine microalgae to replace the unsustainable supply of fishmeal and fish oil. In Thailand alone, which produces about 10% of the current world supply, an investment of $3.0 billion on only 11,100 hectares, roughly 1.5% of the land now dedicated to oil palm production, would yield annual net income of $0.65 billion on sales of $1.0 billion. The global market would be ten times more profitable. If microalgae were used to replace fishmeal and fish oil globally, the effect would be to remove 30% of fishing pressure at the lower end of the food web and would contribute to a restoration of marine ecosystems.

## Electronic supplementary material

Supplementary Information
