# Supplementary material for: Marine microalgae commercial production improves sustainability of global fisheries and aquaculture
Source: Sci Rep. 2018 Oct 10;8:15064. doi: 10.1038/s41598-018-33504-w (PMC6180066; doi:10.1038/s41598-018-33504-w)
Supplement: Supplementary file 1 — Supplementary Information [file 41598_2018_33504_MOESM1_ESM.pdf]

# SUPPLEMENTARY INFORMATION

Marine microalgae commercial production improves sustainability of global fisheries and aquaculture

Colin M. Beal[1,2], Léda N. Gerber[2], Supis Thongrod[3], Wutiporn Phromkunthong[4], Viswanath Kiron[5], Joe Granados[2], Ian Archibald[2,6], Charles H. Greene[2,7], Mark E. Huntley*[2,8]

[1] B&D Engineering and Consulting LLC, 7419 State Hwy 789, Lander, WY 82520, United States

[2] University of Hawaii at Hilo, Pacific Aquaculture & Coastal Resources Center, College of Agriculture, Forestry, and Natural Resource Management, 200 W. Kawili St., HI 95720, United States

[3] Thai Union Feedmill Co., Ltd., 89/1 Moo2, Tambon Kalong, Amphoe Muang, Samutskorn Province, Thailand 74000

[4] Prince of Songkla University, Department of Aquatic Science, Faculty of Natural Resources, Hat Yai, Songkhla Province, Thailand 90112

[5] Nord University, Faculty of Biosciences and Aquaculture, 8049 Bodo, Norway

[6] Cinglas Ltd, Chester, United Kingdom

[7] Cornell University, Department of Earth and Atmospheric Sciences, 4120 Snee Hall, Ithaca, NY 14853, United States

[8] Cornell University, Department of Animal Sciences, 149 Morrison Hall, Ithaca, NY 14853, United States

* Corresponding author. Tel: +1(808) 640-4239, E-mail address: meh333@cornell.edu

Supplementary Table S1. Capital costs for a 111-ha facility in Thailand.

| Item | Cost ($/ha) |
| --- | --- |
| Water Supply Equipment | 27,400 |
| Water Supply/Discharge Piping | 170 |
| PBR System | 1,420 |
| PBR Piping | 5,390 |
| Pond System | 77,100 |
| Pond Piping | 28,800 |
| CO2 Piping | 50 |
| Secondary Settlers (earthworks & liner) | 315 |
| Ring Dryer | 14,300 |
| Filter Press | 10,400 |
| Instrumentation + control | 6,550 |
| Other Piping | 40.0 |
| Electrical | 11,500 |
| Buildings | 30,800 |
| Land preparation | 20,600 |
| Other | 26,000 |
| | |
| Cultivation Total ($/ha) | 261,000 |
| Cultivation Total ($) | 29,000,000 |
| Processing Total ($/ha) | 2,590 |
| Processing Total ($) | 289,000 |
| Total Cost $ | 29,300,000 |

Supplementary Table S2. Labor costs for facility in Thailand

| Thailand | Per Employee | | | | | | |
|---|---|---|---|---|---|---|---|
| | | Base | Fringe | Hourly | Annual | No. | Total |
| Position | Function | ($ h-1) | ($ h-1) | ($ h-1) | ($ yr-1) | FTE | ($000s yr-1) |
| Operations Staff | | | | | | | |
| Harvest technician | Harvest/inoculate ponds | *$1.72* | $0.60 | $2.33 | $4,750 | 17.0 | $80.69 |
| Processing tech | Operate processing eq. | *$2.32* | $0.81 | $3.13 | $6,380 | 3.0 | $19.15 |
| Plant engineer | Plant maintenance | *$3.60* | $1.26 | $4.86 | $9,910 | 5.0 | $49.57 |
| Lab technician | Scale-up + QA/QC | *$3.56* | $1.25 | $4.81 | $9,820 | 10.0 | $98.17 |
| IT engineer | Process control system | *$5.06* | $1.77 | $6.84 | $13,900 | 2.0 | $27.90 |
| Production manager | Operations | *$6.47* | $2.26 | $8.74 | $17,800 | 1.0 | $17.82 |
| Total Operations | | | | | | 38.0 | $293 |
| Administrative Staff | | | | | | | |
| Franchise manager | Overall administration | *$11.76* | $4.12 | $15.88 | $32,400 | 1.0 | $32.40 |
| Sales manager | Sales & marketing | *$8.91* | $3.12 | $12.03 | $24,500 | 1.0 | $24.54 |
| Accounting | Fiscal management | *$7.65* | $2.68 | $10.32 | $21,100 | 0.5 | $10.53 |
| Human resources | Personnel | *$7.65* | $2.68 | $10.32 | $21,100 | 0.5 | $10.53 |
| Total Admin. | | | | | | 3.0 | $78 |
| Personnel Summary | | | | | | | |
| Total Operations | | | | | | 38 | $293 |
| Total Administration | | | | | | 3 | $78 |
| Total Personnel | | | | | | 41 | $371 |

Supplementary Table S3. Key parameters for the techno-economic analysis.

| Parameter | Value |
|---|---|
| Discount rate | 10% |
| Tax rate | 20% |
| Capital | $29.3M |
| Equity | 40% |
| Interest rate | 8% |
| Loan term | 10 years |
| Depreciable investment | 89% ($26.0M) |
| Maintenance rate (of capital) | 1% per year |
| Insurance rate (of capital) | 1% per year |
| Land rent (scaled to Thailand)[1] | $11.89/ha-yr |
| MACRS % depreciation[2], years 1-8 | 14.3, 24.5, 17.5, 12.5, 8.9, 8.9, 8.9, 4.5 |
| Facility life | 30 years |
| Algal biomass meal baseline market value[3] | $1,400/MT |
| Algal lipids baseline market value[3] | $1,800/MT |

Supplementary Table S4: Probability distribution functions

| **Parameter** | **Distribution law** | **mode** | **min** | **max** | **mu** | **sigma** |
|---|---|---|---|---|---|---|
| Productivity [g/m$^2$-day] | Normal | | | | 23.4 | 4.48 |
| Lipid fraction [-] | Normal | | | | 0.37 | 0.07 |
| Stoichiometric CO2 [g-$CO_2$/g-algae] | Triangular | 1.78 | 1.23 | 1.88 | | |
| Efficiency of CO2 absorption [-] | Uniform | | 0.5 | 0.95 | | |
| Capacity factor [-] | Triangular | 0.90 | 0.80 | 0.98 | | |
| Electricity dewatering [kWh/m$^3$] | Triangular | 0.30 | 0.18 | 0.43 | | |
| Oil extraction efficiency [-] | Uniform | | 0.70 | 0.95 | | |
| Ammonia price [$/tonne] | Normal | | | | 455 | 76.0 |
| DAP price [$/tonne] | Normal | | | | 410 | 60.0 |
| Electricity price [$/kWh] | Lognormal | | | | -2.65 | 0.25 |
| Error on investment costs [%] | Uniform | | -30% | 30% | | |
| Algal oil price [$/tonne] | Normal | | | | 1.80 | 0.15 |
| Lipid-extracted algae price [$/tonne] | Normal | | | | 1.40 | 0.05 |
| Geographic cost modifier [-] | Triangular | 0.58 | 0.25 | 1.00 | | |
| $CO_2$ price [$/kg] | Triangular | 0.075 | 0.040 | 0.160 | | |
| PBR plastic cost [%] | Uniform | | -30% | +30% | | |
| Heat required for extraction [%] | Uniform | | -30% | +30% | | |
| Error on labor costs [%] | Uniform | | -30% | +30% | | |

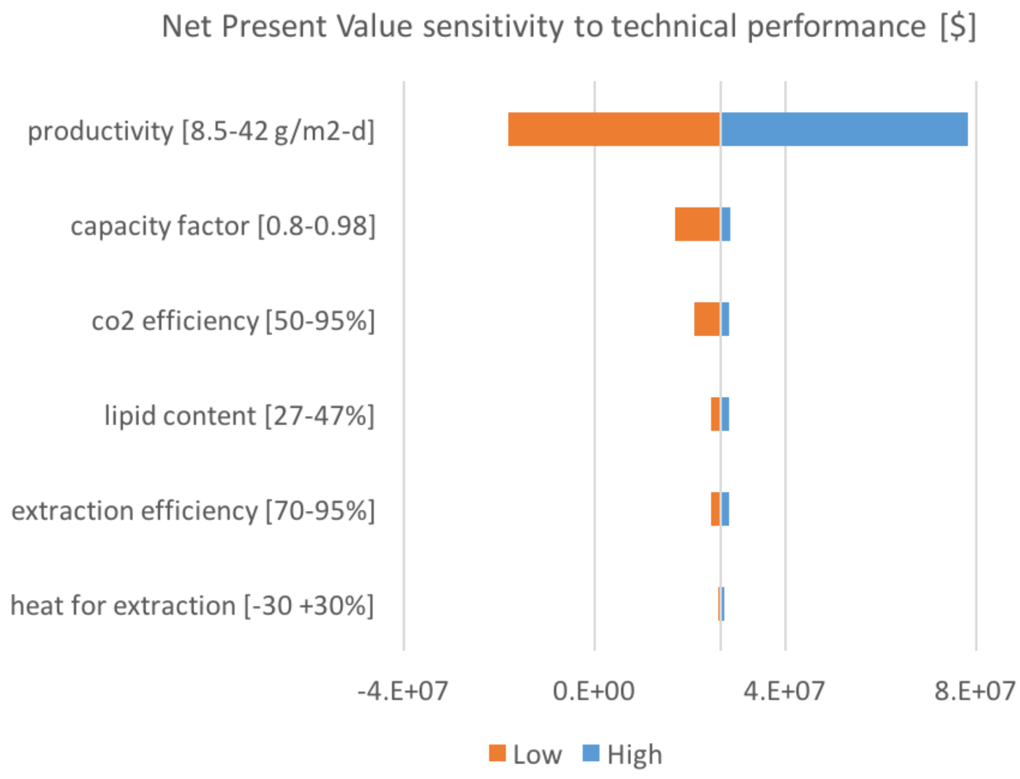


Supplementary Figure S1: Results of the sensitivity analysis for the Net Present Value (NPV) indicator.

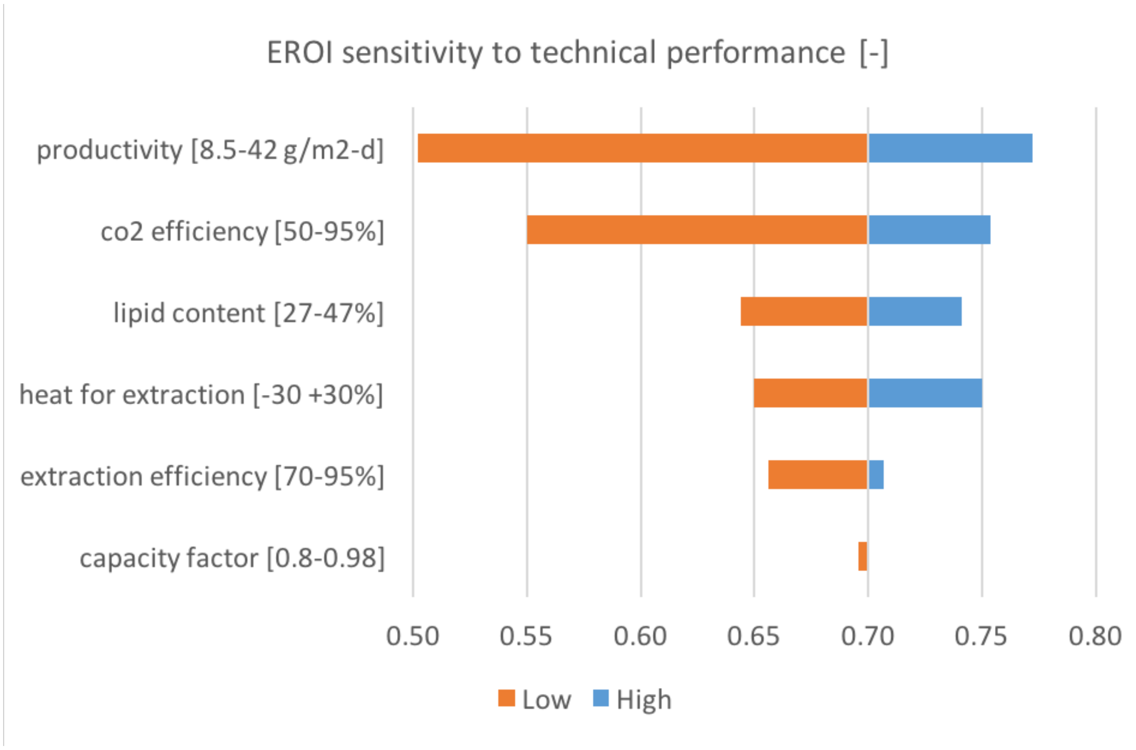


Supplementary Figure S2: Results of the sensitivity analysis for the energy return on investment (EROI) indicator.

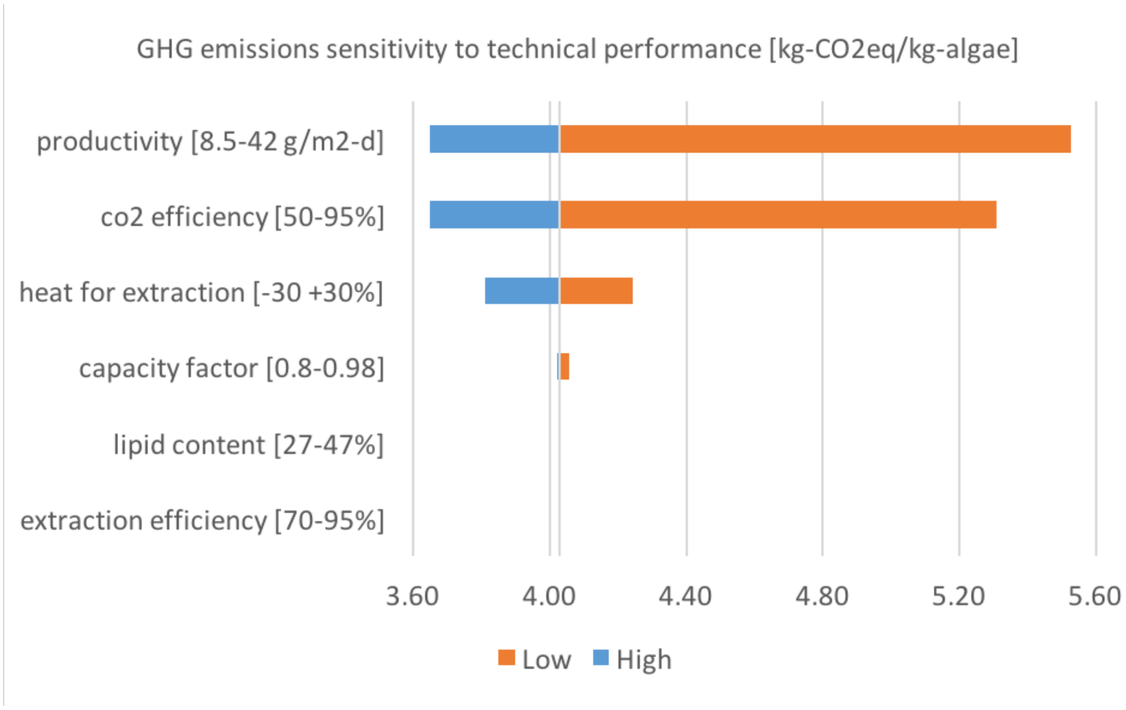


Supplementary Figure S3: Results of the sensitivity analysis for the greenhouse gases (GHG) emissions indicator.

Supplementary Information References


1. USDA. Land Values, 2016 Summary, National Agricultural Statistics Service, ISSN: 1949-1867, 2016
2. IRS. *Publication 946 (2013), How to Depreciate Property*, 2013
3. FAO. The State of World Fisheries and Aquaculture 2016. pp. 200, Rome, 2016
